# Deletion of hepatic small heterodimer partner ameliorates development of nonalcoholic steatohepatitis in mice

**DOI:** 10.1016/j.jlr.2023.100454

**Published:** 2023-10-10

**Authors:** Yoon-Kwang Lee, Jung Eun Park, Mikang Lee, Ryan Mifflin, Yang Xu, Robert Novak, Yanqiao Zhang, James P. Hardwick

**Affiliations:** 1 Department of Integrative Medical Sciences, College of Medicine, Northeast Ohio Medical University, Rootstown, OH, USA; 2 Department of Pathology, College of Medicine, Northeast Ohio Medical University, Rootstown, OH, USA

**Keywords:** cholesterol, cholesterol 7-alpha hydroxylase, inflammation, lipoproteins, liver, mitochondria

## Abstract

Small heterodimer partner (SHP, *Nr0b2*) is an orphan nuclear receptor that regulates bile acid, lipid, and glucose metabolism. *Shp*^*−/−*^ mice are resistant to diet-induced obesity and hepatic steatosis. In this study, we explored the potential role of SHP in the development of nonalcoholic steatohepatitis (NASH). A 6-month Western diet (WD) regimen was used to induce NASH. *Shp* deletion protected mice from NASH progression by inhibiting inflammatory and fibrotic genes, oxidative stress, and macrophage infiltration. WD feeding disrupted the ultrastructure of hepatic mitochondria in WT mice but not in *Shp*^*−/−*^ mice. In *ApoE*^*−/−*^ mice, *Shp* deletion also effectively ameliorated hepatic inflammation after a 1 week WD regimen without an apparent antisteatotic effect. Moreover, *Shp*^*−/−*^ mice resisted fibrogenesis induced by a methionine- and choline-deficient diet. Notably, the observed protection against NASH was recapitulated in liver-specific *Shp*^*−/−*^ mice fed either the WD or methionine- and choline-deficient diet. Hepatic cholesterol was consistently reduced in the studied mouse models with *Shp* deletion. Our data suggest that *Shp* deficiency ameliorates NASH development likely by modulating hepatic cholesterol metabolism and inflammation.

Nonalcoholic fatty liver disease (NAFLD) is characterized by an increase in hepatic fat concentration (steatosis) with or without inflammation and fibrosis. The presence of inflammation in steatosis is the hallmark of the serious chronic liver disorder, nonalcoholic steatohepatitis (NASH). Subsequent fibrotic insults lead to cirrhosis and hepatocellular carcinoma. Although the specific underlying mechanism is not fully understood, the prevalence of metabolic disorders, such as obesity and diabetes in modern societies, is strongly associated with the NAFLD pandemic. Development of NAFLD is initiated by relatively benign hepatic steatosis, abnormal liver fat accumulation (exceeding 5% fat by liver weight), which affects one-third of the US population (1). Even though fat accumulation is a prerequisite for the development of NASH, only a fraction (<30%) of subjects with fatty liver progress to develop NASH. The current prevailing dogma for NASH pathogenesis is the “two-hit theory” (2, 3). The first hit involves abnormal fat accumulation, and the second hit involves oxidative stress, inflammation, dietary factors, or genetic susceptibility (4). Currently, it is unclear how the second hit is initiated in NAFLD. Only a small portion of people with steatosis eventually develop NASH or the end stage of NAFLD; thus, abnormal fat accretion in the liver does not simply result in the development of NASH.

The orphan nuclear hormone receptor SHP plays important roles in bile acid (BA), lipid, and glucose homeostasis (5, 6, 7, 8, 9, 10, 11, 12). Some critical metabolic results have been obtained from *Shp*-null and transgenic mouse studies, which demonstrated that deletion of *Shp* protects animals from diet-induced obesity and hepatic steatosis and that its hepatic overexpression reverses the phenotype (10, 13, 14). A few studies also reported that deletion of *Shp* reduced not only hepatic lipid accumulation but also inflammation and liver damage caused by diet (15, 16). SHP has also been reported to function as a transcriptional coactivator of NF-κB, a key transcription factor for immune and inflammatory responses (17). However, Yuk *et al.* (18) reported that *Shp*-null mice exhibited augmented-inflammatory responses to lipopolysaccharide challenge, thus leading to a higher mortality rate. Myronovych *et al.* (19) also showed that *Shp*-null mice on a high-fat diet had a proinflammatory hepatic phenotype. In recent studies, liver-specific SHP overexpression or deletion ameliorated or worsened NASH development induced by a diet containing high fat, high cholesterol, and high fructose (20, 21). These studies claimed that the protection was mediated by suppression of inflammatory chemokine (C-C motif) ligand 2 transcription and inhibition of nuclear translocation of NF-κB by SHP.

In our previous studies, we suggested several molecular mechanisms underlying the modified hepatic fat accumulation by *Shp* gene deletion (10, 22). Gene expression analysis using a BeadChip array revealed that many genes involved in inflammation and fibrosis were also downregulated in the livers of *Shp*^*−/−*^ mice (22). In this study, we explored the role of SHP in the development of diet-induced NASH using whole-body and liver-specific *Shp*-null mice. Two different diets were utilized to induce NASH; a Western diet (WD) as described in a previous report (22) and a methionine- and choline-deficient (MCD) diet. In addition, the *Shp*-null allele was introduced into *ApoE*-null background mice to generate *ApoE/Shp* double knockout (*ApoE/Shp*^−/−^) mice. *ApoE*-null mice have been known to develop hepatic inflammation without fat accumulation upon short-term WD feeding. This mouse model provides insight into the direct role of SHP in the second hit, the inflammatory response, apart from hepatic fat accumulation (23).

## Materials and Methods

### Mice and diet

The congenic *Shp*^*−/−*^ mice used in this study have been described in previous studies (10, 22). To generate congenic C57BL/6 mice with liver-specific SHP deletion (*LvShp*^*−/−*^), we back-crossed floxed *Shp* (*Shp*^*flox/flox*^) mice with C57BL/6NHsd mice (Envigo, IN) for 10 generations (16). The congenic *Shp*^*flox/flox*^ mice were then crossed with albumin promoter-driven cyclization recombinase (Cre) mice (*Alb-Cre*, B6.Cg-*Speer6-ps1*^*Tg(Alb-cre)21Mgn*^/J; Jackson Laboratory) to obtain *LvShp*^*−/−*^ mice. To generate *ApoE*/*Shp* double knockout mice (*ApoE/Shp*^*−/−*^), whole-body *Shp* deletion was introduced into the backcrossed apolipoprotein E-deficient (*ApoE*^*−/−*^, B6.129P2-*Apoe*^*tm1Unc*^/J; Jackson Laboratory) mice against C57BL/6NHsd for five to six generations. For control mice, age-matched C57BL/6, *ApoE*^*−/−*^, and *Shp*^*flox/flox*^ or *Alb*-*Cre* mice were used against *Shp*^*−/−*^, *ApoE/Shp*^*−/−*^, and *LvShp*^*−/−*^ mice, respectively. Throughout the experiments, only male mice were used and maintained in the accredited pathogen-free facility at Northeast Ohio Medical University with a 12 h light–dark cycle. The WD containing high cholesterol (0.15% cholesterol and 42% fat) (10) and MCD diet were purchased from Envigo (Indianapolis, IN, WD; TD.88137, MCD; TD.90262). *Shp*^*−/−*^, *LvShp*^*−/−*^, and their respective control mice were fed the WD for 6 months or MCD for 1 month starting at 2 months of age to induce NAFLD and NASH, respectively. For the MCD diet regimen, a defined amino acid diet (TD.94149; Envigo) was used as the control diet, and for the WD regimen, a regular chow (14.8% [w/w] total fat [16.97% kcal from fat and 233 ppm cholesterol]) was obtained from Formulab Diet (Fort Worth, TX). *ApoE/Shp*^*−/−*^ and *ApoE*^*−/−*^ mice were fed the WD for 1 week to induce hepatic inflammation without noticeable fat accumulation (23). Mice were euthanized to collect tissues after overnight fasting. All animal care and use protocols were approved by the Institutional Animal Care and Use Committee of Northeast Ohio Medical University.

### Hepatic lipid measurement and tissue staining

Hepatic lipid extraction has been described previously (24). Triglyceride (TG) and cholesterol concentrations were determined by commercial kits (Thermo Fisher Scientific, Rockford, IL) and presented by milligram per gram of wet liver weight. Sirius red staining was performed with liver sections using a commercial kit (Polysciences, Inc, Warrington, PA). H&E staining and Oil Red O staining have been described previously (22). The stained liver sections were evaluated for NAFLD activity scores by a certified pathologist (Dr Novak) in a blinded manner. Immunofluorescence was performed with sectioned frozen liver tissues using AlexaFluor® 750-conjugated anti-mouse F4/80 monoclonal antibody (Thermo Fisher Scientific).

### Mitochondrial citrate synthase activity

Citrate synthase activity was determined as previously described with slight modification (25). First, 100 mg of liver was homogenized in 1 ml of STE buffer (250 mM sucrose, 10 mM Tris-HCl, and 1 mM EDTA) and centrifuged at 800 *g* for 10 min. Citrate synthase activity was measured by mixing the supernatant (10 μl) with 40 μl of a buffer containing 0.575% Triton X-100, 0.75 mM acetyl-CoA, 0.25 mM 5,5′-dithiobis(2-nitrobenzoic acid), 0.25 M triethanolamine, 2 mM EDTA, and 1 M Tris-HCl, followed by addition of 50 μl of 1 mM oxaloacetate to initiate the reaction. The change in absorbance at 412 nm was recorded every 30 s over a 5 min period using Synergy 4 (BioTek, Winooski, VT) at 37°C. The activity was calculated by comparing the slope from the linear portion of the curve with the oxaloacetate standard curve slope and normalized to the protein concentration.

### Mitochondrial DNA content

A piece of liver (50 mg) was homogenized in 500 μl of mitochondria isolation buffer containing 200 mM trehalose, 68 mM sucrose, 10 mM Hepes (pH 7.4), 10 mM KCl, 1 mM EDTA, and 1 mM EGTA. Homogenates (100 μl) were mixed with 0.5 ml of cetyltrimethyl ammonium bromide DNA isolation buffer as previously described (26). Then, 20 ng of isolated DNA was used to amplify the 438 nucleotides of the mitochondrial 12S rRNA gene using a forward primer (5′-CAAACTGGGATTAGATACC-3′) and reverse primer (5′-GAGGGTGACGGGCGGTGTGT-3′). The amplified values were normalized to globin gene (forward: 5′-GTTCCACCCGCCTCACATTG-3′; reverse: 5′-ACAGATGGAGCGTCCAGAAAG-3′) amplification values.

### Measurement of hepatic lipid peroxidation and production of reactive oxygen species

Hepatic lipid peroxidation was analyzed by quantification of malondialdehyde using a commercial kit measuring thiobarbituric acid reactive substances (Cayman Chemical, Ann Arbor, MI), as described previously (27). The obtained malondialdehyde concentrations were normalized using protein concentrations. For measurement of hepatic reactive oxygen species (ROS) production, overnight fasting mice were injected intraperitoneally with dihydroethidium (50 mg/kg). The mice were euthanized 3 h after injection to collect their livers. Homogenized liver tissues were processed to measure the fluorescent 2-hydroxyethidium levels using Synergy 4. Fluorescent units are presented per wet liver weight.

### Transmission electron microscopy

We analyzed the ultrastructure of hepatocytes from WD-fed mice using a transmission electron microscope (JEM-100S; JEOL USA, Peabody, MA) in the NEOMED core facility. Briefly, the mice were perfused with 2% glutaraldehyde in 0.1 M PBS, and their livers were fixed with the same solution for 2 h at room temperature. The fixed livers were sequentially dehydrated with ethanol and propylene oxide and infiltrated with Embed 812 resin to produce a final block after baking overnight at 70°C.

### Quantitative real-time PCR

Total RNA was isolated from livers of overnight-fasted mice using Trizol reagent (Thermo Fisher Scientific). Complementary DNA was synthesized from the isolated RNA using PrimeScript RT Master Mix (Takara Bio USA, Mountain View, CA). SYBR Green was used to perform RT–quantitative PCR (qPCR) analysis on the CFX96 system (Bio-Rad, Hercules, CA). *Gapdh* expression was used to normalize target gene expression.

### Determination of plasma alanine aminotransaminase and aspartate aminotransaminase values

Alanine aminotransaminase and aspartate aminotransaminase activities were determined via the colorimetric method using commercial kits (Thermo Fisher Scientific).

### Plasma lipoprotein analysis

The plasma lipoprotein profile was determined using the BioLogic DuoFlow QuadTec FPLC system (Bio-Rad Laboratories, Hercules, CA), as previously described (28). TG and cholesterol in the fractionated samples were quantified using commercial kits (Thermo Fisher Scientific).

### Statistical analysis

Data are presented as means ± SEM if not otherwise specified. All the *P* values were obtained using Student’s *t*-test between the indicated two groups. Sample numbers per group were between 5 and 15. *P* < 0.05 was designated as statistically significant. One symbol (# or ∗) represents *P* < 0.05, two symbols represent *P* < 0.01, and three symbols represent *P* < 0.005. We also used two-way ANOVA on various datasets using GraphPad Prism software (GraphPad Software, Inc). The results are presented in the associated figures.

## Results

### *Shp* deletion protects mice from diet-induced inflammatory response

**Fig. 1:**
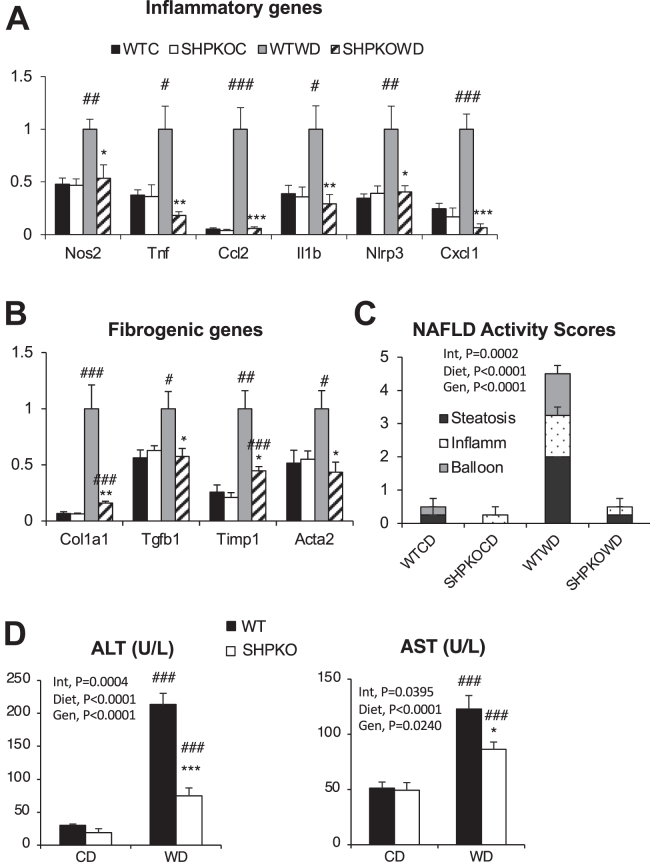
*Shp*^*−/−*^ mice were protected from the development of hepatic inflammation and fibrosis induced by WD feeding. Gene expression in livers of WT and *Shp*^*−/−*^ mice fed control diet (CD) or WD for 6 months was determined by qPCR analysis. Hepatic mRNA levels of genes involved in inflammation (A) and fibrosis (B) were presented with means ± SEM (*n* = 5–6). C: Blindly evaluated NAFLD activity scores were plotted with steatosis, inflammation, and ballooning scores from liver sections stained with H&E (*n* = 4). D: ALT and AST values were presented as means ± SEM. Statistics were obtained from both two-way ANOVA and Student’s *t*-test (*n* = 7–8). #comparison with CD, ∗comparison with WT counterparts. Three symbols represent *P* < 0.005, two symbols *P* < 0.01, and one symbol *P* < 0.05. ALT, alanine aminotransferase; AST, aspartate aminotransferase.

We previously demonstrated that whole-body deletion of orphan nuclear hormone receptor *Shp* protects mice from diet-induced obesity but exacerbates the diabetic phenotype (10). In a follow-up study, we identified that the livers of the mice had reduced fat accumulation with a novel transcriptional cascade involving various nuclear hormone receptors (22). Upon examining the BeadChip array data obtained from the livers of WT and *Shp*^*−/−*^ mice in this study, we discovered that many genes involved in inflammation and fibrosis were also significantly downregulated in the livers of *Shp*^*−/−*^ mice fed WD compared with those of WT counterparts, with significant upregulation of those genes after 6 months of the WD regimen (supplemental Table S1). This strongly suggests that SHP plays a role in the development of NASH. To investigate further, livers of WT and *Shp*^*−/−*^ mice fed the WD for 6 months were processed to analyze inflammatory and fibrotic gene expression using RT–qPCR analysis (Fig. 1). As observed in the BeadChip array, mRNA expression of important genes occurred in inflammatory pathways, such as expression of inducible nitric oxide synthase (*Nos2*), tumor necrosis factor-alpha (*Tnf*), monocyte chemoattractant protein 1 (*Ccl2*), interleukin-1-beta (*Il1b*), NLR family pyrin domain containing 3 (*Nlrp3*), and chemokine (C-X-C motif) ligand 1 (*Cxcl1*) (Fig. 1A), and in fibrogenesis, such as expression of collagen type I alpha 1 (*Col1a1*), transforming growth factor beta 1 (*Tgfb1*), tissue inhibitor of metalloproteinase 1 (*Timp1*), and alpha-smooth muscle actin (*Acta2*) (Fig. 1B). These were strongly upregulated in the livers of WT mice and almost completely blunted in *Shp*^*−/−*^ livers upon WD feeding. The NAFLD activity scores from liver H&E sections evaluated by a pathologist in a blind study agreed with the gene expression pattern (Fig. 1C and supplemental Fig. S1A). Serum alanine aminotransaminase and aspartate aminotransaminase levels were also lower in *Shp*^*−/−*^ mice than in WT mice upon WD challenge (Fig. 1D).

### Reduced oxidative stress was manifested in *Shp*^*−/−*^ mice fed the WD

**Fig. 2:**
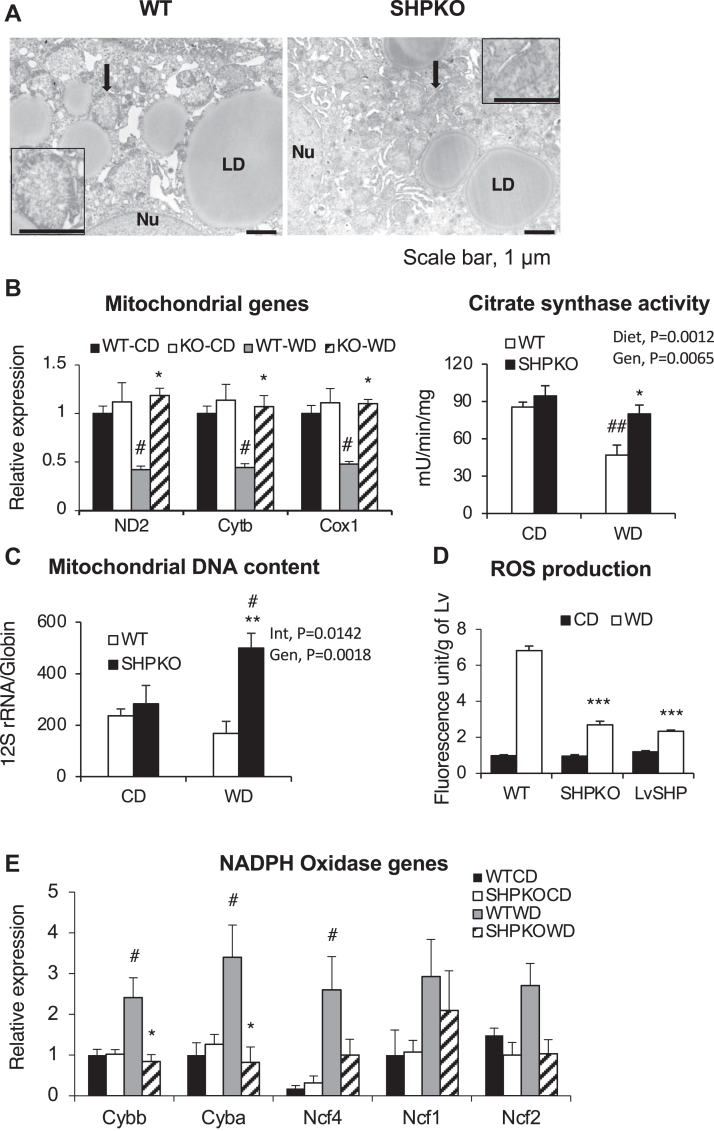
Hepatic mitochondrial dysfunction and ROS production were ameliorated in SHP-null mice fed WD. A: Representative electron micrographs of WT and *Shp*^*−/−*^ mice livers are depicted. Arrow points to mitochondrium, whose image is magnified in an inset. The scale bar represents 1 μm. B: Expression of mitochondrial-encoded genes from livers of WT and *Shp*^*−/−*^ mice fed control diet (CD) or WD (*left panel*). Mitochondrial-specific citrate synthase activities were quantified from liver extract as described in the Methods section (*right panel*) (*n* = 5–6). C: relative mitochondrial DNA content was presented by PCR amplification units of mitochondrial-specific 12 rRNA genes after normalization with nuclear-specific globin gene amplification (*n* = 6). D: hepatic ROS production was presented by fluorescence intensity per wet liver weight after dihydroethidium injection as described in the Materials and methods section (*n* = 4–7). E: hepatic expression of NADPH oxidase genes in WT and *Shp*^*−/−*^ mice (*n* = 5–6). Cox1, cytochrome *c* oxidase subunit 1; Ctyb, cytochrome *b*; LD, lipid droplet; ND2, NADH dehydrogenase subunit 2; Nu, nucleus.

Oxidative stress has been strongly implicated in the development of NASH (29, 30). The stress is mediated by free radicals, ROS, and enzymes, such as lipoxygenase, cyclooxygenase, cytochrome P450 monooxygenases, and NADPH oxidases (30). The most important cellular organelle responsible for ROS production is mitochondria. Mitochondrial dysfunction manifests as a hallmark of NASH pathology (31, 32, 33). Therefore, we assessed several biochemical and molecular properties of mitochondria in the WD-fed groups using transmission electron microscopy, RT–qPCR analysis, mitochondrial enzyme activity, mitochondrial DNA copy numbers, and ROS production rate. The transmission electron microscopy image from the WD-fed WT mouse liver clearly showed lipid droplet accumulation and swollen mitochondria with loss of well-defined cristae, a characteristic of the NASH phenotype (34), whereas mitochondria from *Shp*^*−/−*^ liver maintained normal morphology even in the presence of adjacent lipid droplets (Fig. 2A). In agreement with this, mRNA expression of mitochondrial genes and citrate synthase activity were strongly attenuated in the livers of WT mice but remained normal in the livers of *Shp*^*−/−*^ mice in the WD-fed group (Fig. 2B). Interestingly, mitochondrial DNA copy numbers per nuclear DNA were significantly increased in the livers of WD-fed *Shp*^*−/−*^ mice compared with the livers of WT counterparts and control diet-fed animals (Fig. 2C). In addition, a marked increase in ROS production was observed in the livers of WT mice fed the WD, which was significantly reduced in the *Shp*^*−/−*^ livers (Fig. 2D). We also assessed expression of NADPH oxidase subunit genes using RT–qPCR. NADPH oxidase is involved in an important host cell defense against bacterial infections by producing ROS, which can simultaneously induce intracellular damage and is considered an important contributor to the development of NASH and hepatic fibrogenesis (35). As shown in Fig. 2E, WT mice displayed a strong initiation of mRNA expression of NADPH oxidase genes upon WD challenge, whereas deletion of *Shp* significantly abrogated the upregulation of this expression. Interestingly, our earlier BeadChip array analysis showed that mRNA expressions of many antioxidant genes were downregulated, whereas mitochondrial thioredoxin (*Txn2*) expression remained higher in WD-fed *Shp*^*−/−*^ mice than in the WT mice (supplemental Table S2). These results indicate that reduced ROS production from mitochondria may play a role at least partially in minimizing liver damage from WD challenge in *Shp*^*−/−*^ mice.

### Deletion of *Shp* attenuates the hepatic inflammatory response to the 1-week WD challenge in *ApoE*-null mice without noticeable fat accumulation

**Fig. 3:**
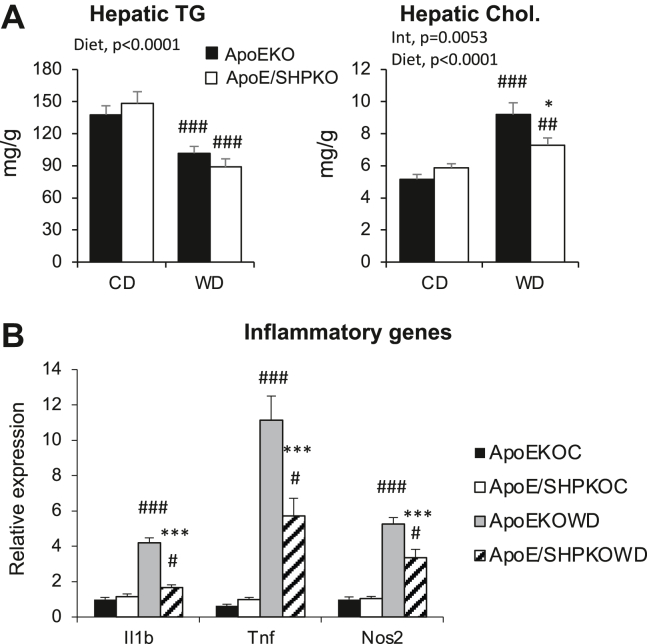
One-week WD challenge was performed with *ApoE*^*−/−*^ and *ApoE/Shp*^*−/−*^ mice to measure hepatic inflammation. Two-month-old *ApoE*^*−/−*^ and *ApoE/Shp*^*−/−*^ mice were fed control diet (CD) or WD for 1 week. After the regimen, livers were analyzed for lipid accumulation and the expression of inflammatory genes. A: Hepatic TG and cholesterol levels were quantified and presented as means ± SEM (*n* = 5–6). B: The mRNA levels of major inflammatory genes were assessed using qPCR analysis and presented as means ± SEM (*n* = 5–7).

In the proposed two-hit theory for the development of NASH, the second hit was represented by inflammation initiated after the first hit of fat accumulation (2, 3). In earlier studies, *ApoE*-null mice were resistant to diet-induced hepatic steatosis and prone to develop inflammation in the short term upon high fat and high cholesterol diet challenge (23, 36, 37). Therefore, *ApoE*-null mice with *Shp* gene deletion were generated and challenged with the short-term WD to assess whether the reduced inflammatory responses in *Shp*^*−/−*^ livers are related to reduced fat accumulation or the intrinsic effect of SHP deletion regardless of fat concentration. As shown in Fig. 3, 1 week of WD feeding was sufficient to induce inflammatory responses (panel B) without any TG accumulation (panel A & supplemental Fig. S1B) in the livers of the *ApoE*-null mice. However, *Shp* deletion in *ApoE*-null (*ApoE/Shp*^*−/−*^) mice blunted the inflammatory response without significant change in fat accumulation, indicating that reduced hepatic inflammation is caused by *Shp* deletion and is not secondary to the lower TG accumulation. This result strongly suggests that SHP regulates hepatic inflammatory pathways independent of TG accumulation; thus, understanding the regulation may help determine the underlying mechanism of NASH development.

### The increase in cholesterol upon WD feeding is attenuated in *Shp*^*−/−*^ and *ApoE/Shp*^*−/−*^ mice

**Fig. 4:**
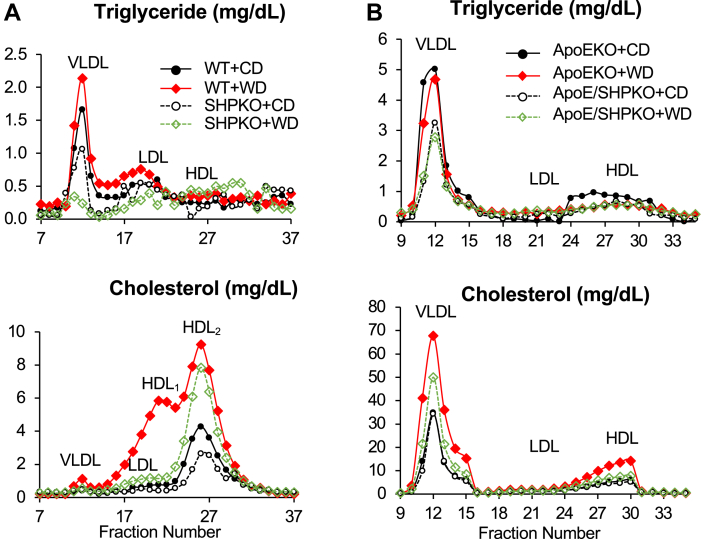
Plasma lipoprotein analysis was performed with WT, *Shp*^*−/−*^, *ApoE*^*−/−*^, and *ApoE/Shp*^*−/−*^ mice on WD regimen. A: Fractionated plasma samples (combined from five mice each) from WT and *Shp*^*−/−*^ mice fed control diet (CD) or WD were analyzed for their TG and cholesterol levels. Their concentrations were plotted with fractionated sample numbers (*X*-axis). Major lipoprotein classes were indicated in the graph. B: The plots obtained from plasma samples of *ApoE*^*−/−*^ and *ApoE/Shp*^*−/−*^ mice (*n* = 5). The cholesterol curves of *ApoE*^*−/−*^ and *ApoE/Shp*^*−/−*^ fed CD almost overlap in the bottom graph of panel B.

SHP regulates the expression of the *Cyp7a1* gene, which encodes a rate-limiting enzyme in the conversion of cholesterol to BAs. *Shp* deletion lowers hepatic and serum cholesterol levels upon WD feeding likely owing to upregulation of *Cyp7a1* gene expression (10, 16). We assessed the plasma lipid profiles using fast protein liquid chromatography to explore their association with the observed liver phenotype in the *Shp*-deleted mice. Although WD feeding increased LDL and HDL fractions significantly in the WT mice, it failed to change the lipoprotein profile in the *Shp*^*−/−*^ mice (supplemental Fig. S2A). When we analyzed TG and cholesterol contents in each fraction, the *Shp*^*−/−*^ mice exhibited lower TG contents in VLDL fractions and lower HDL cholesterol contents than WT mice who were fed the regular chow (Fig. 4A). In WT mice, WD feeding significantly increased the TGs in the VLDL and LDL fractions and cholesterol in the LDL and HDL fractions. Interestingly, in *Shp*^*−/−*^ mice, WD feeding reduced the TG content in the VLDL fraction and increased TG and cholesterol in the HDL fraction. Although 1 week of WD feeding considerably increased the VLDL fraction in *ApoE*^*−/−*^ mice, the increase was blocked by *Shp* deletion; thus, a change in the lipoprotein profile was not observed in the *ApoE/Shp*^*−/−*^ mice upon WD feeding (supplemental Fig. S2B). As observed in the WT mice, *Shp* deletion in *ApoE*^*−/−*^ mice decreased the TG content but not cholesterol in the VLDL fraction in those fed the regular chow (Fig. 4B). Although 1 week of WD feeding did not affect the TG profiles considerably in either genotype, it increased the cholesterol content in the VLDL and HDL fractions of *ApoE*^*−/−*^ mice compared with those of *ApoE/Shp*^*−/−*^ mice. This result agrees with the previously suggested role of cholesterol in NASH development (31, 38, 39).

### Deletion of *Shp* ameliorates the NASH phenotype induced by the MCD diet

**Fig. 5:**
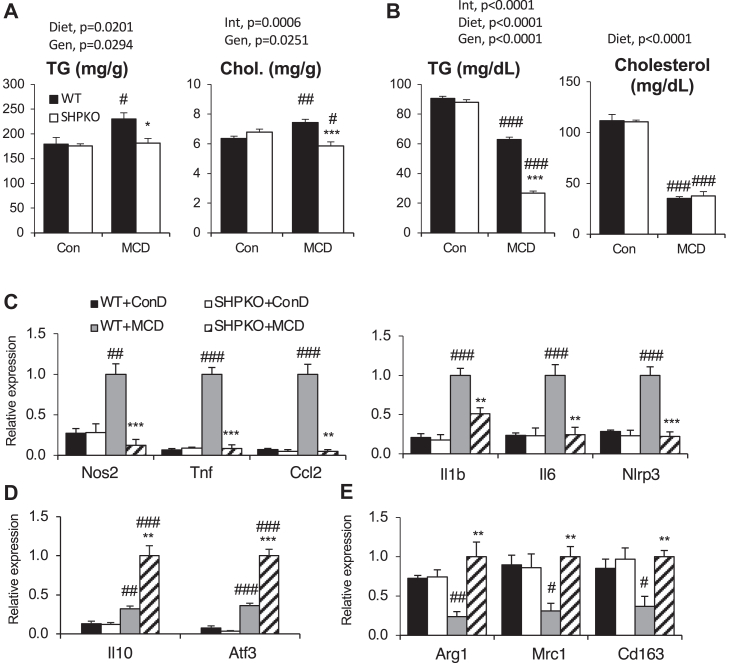
Hepatic lipid and inflammatory gene expression were assessed with WT and *Shp*^*−/−*^ mice after 1-month MCD diet regimen. The 2-month-old experimental mice were fed amino acid-defined control diet (Con) or MCD diet for 1 month. Livers and blood were collected at the end of regimen after overnight fasting. A: Hepatic TG and cholesterol levels were presented after normalization by wet liver weight. B: Plasma levels of TG and cholesterol from the mice. Expression of hepatic genes involved in inflammation (C), anti-inflammation (D), and M2 macrophage markers (E) was presented as means ± SEM (*n* = 5). Two-way ANOVA results were shown with *P* values. Gen, genotype; Int, interaction.

**Fig. 6:**
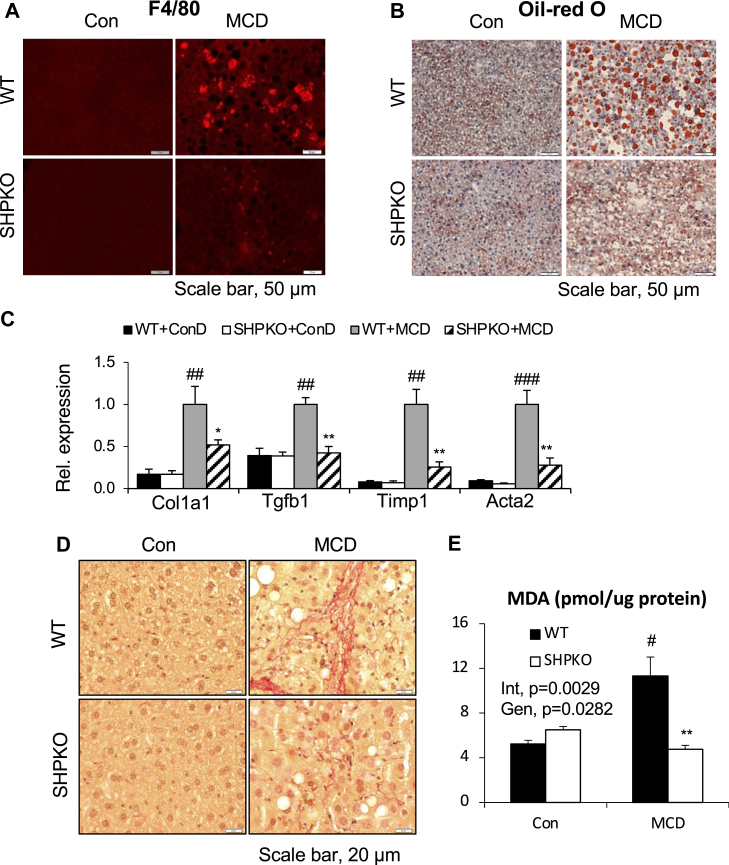
Amelioration of hepatic fibrogenesis was evident in the *Shp*^*−/−*^ mice fed MCD diet. Livers and hepatic mRNAs collected for Fig. 5 were processed to analyze the following. A: Liver sections were processed for Oil Red O staining. B: Immunofluorescence was performed with liver sections to assess macrophage infiltration. C: qPCR analysis was performed to quantify the mRNA levels of the indicated fibrogenic genes. D: Sirus red staining was carried out to configure fibrosis with the liver sections. E: TBARS assay was performed with liver samples using a commercial kit. Malondialdehyde contents were normalized by protein amount. Two-way ANOVA and/or Student’s *t*-test were performed to evaluate statistical significance. *P* values are as indicated for Fig. 1. TBARS, thiobarbituric acid reactive substances.

The MCD diet has been frequently used to study NASH in rodent models. We in addition investigated the potential role of SHP in the development of NASH induced by the MCD diet. As previously reported (40), the MCD diet increased hepatic TG and cholesterol concentrations and decreased the plasma concentrations in WT mice by disruption of VLDL secretion (Fig. 5A, B). Although the negative impact on plasma values remained consistent or increased, the positive hepatic impact of the diet was almost completely absent in the *Shp*^*−/−*^ mice. In agreement with an earlier report (41), the MCD diet strongly induced mRNA expressions of inflammatory and M1 macrophage marker genes (Fig. 5C). As observed in the WD regimen, the expressions of these genes were almost completely reduced in *Shp*^*−/−*^ mice. In contrast, the mRNA expressions of the major anti-inflammatory cytokine (interleukin 10) and transcription factor (activating transcription factor 3) were more strongly induced in *Shp*^*−/−*^ mice (Fig. 5D). The mRNA levels of M2 macrophage marker genes, such as arginase (*Arg1*), mannose receptor C type 1 (*Mrc1*), and CD163 (*Cd163*), were significantly attenuated in WT mice by the MCD diet but remained unchanged in the *Shp*^*−/−*^ mice (Fig. 5E). We also assessed inflammatory responses via immunofluorescence analysis. As shown in Fig. 6A, a marked increase in F4/80-positive cells was manifested in the livers of the WT mice after the MCD diet challenge. In agreement with the RT–qPCR results, the increase was almost completely absent in the livers of *Shp*^*−/−*^ mice fed the MCD diet. As evidenced by the biochemical analysis, Oil Red O staining confirmed diminished hepatic steatosis in the *Shp*^*−/−*^ mice compared with that in the WT mice (Fig. 6B). We also analyzed the progression of fibrogenesis in the mice fed the MCD diet. One month of the MCD diet challenge induced mRNA expression of various fibrogenic genes and exhibited strongly positive Sirius red staining in the livers of the WT mice (Fig. 6C, D). As observed for the 6 month WD feeding, the *Shp*^*−/−*^ mice were also protected from MCD diet-induced lipid peroxidation, as assessed by the production of malondialdehyde (Fig. 6E).

### Liver-specific SHP is responsible for the NASH phenotype

**Fig. 7:**
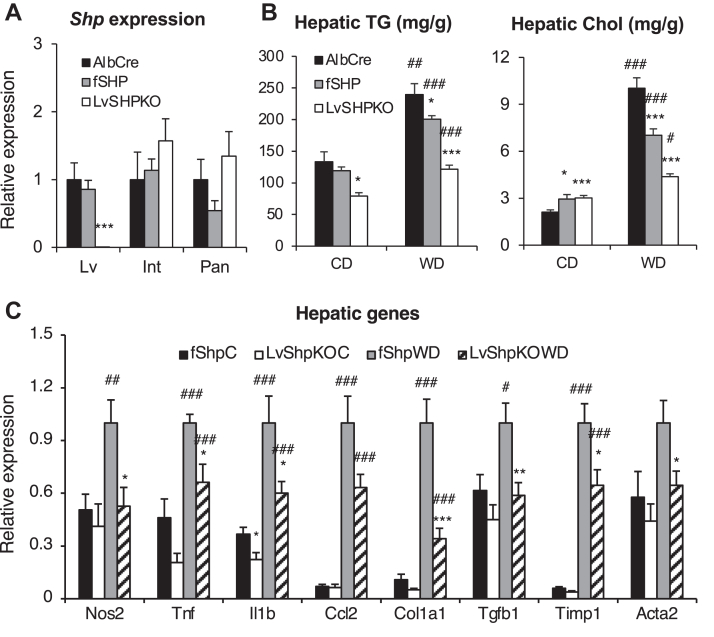
Reduced NASH phenotypes were demonstrated in the liver-specific *Shp*-null (*LvShp*^*−/−*^) mice after 6 months of WD regimen. Albumin-Cre (*AlbCre*) mice, *Shp*^*flox/flox*^ (*fShp*) mice, and liver-specific SHP-null (LvShpKO) mice were challenged with 6 months of WD feeding. A: Liver-specific deletion of *Shp* gene was assessed with different tissues using qPCR analysis. ∗∗∗*P* < 0.005 compared with both *fShp* and *AlbCre* mice. B: Hepatic TG contents were measured with all three groups of mice. C: Expression of hepatic inflammatory and fibrogenic genes from *AlbCre* mice and LvShpKO mice was measured using qPCR analysis (*n* = 5–6). #comparison with control diet (CD), ∗comparison with AlbCre counterparts. Three symbols represent *P* < 0.005, two symbols *P* < 0.01, and one symbol *P* < 0.05. Int; intestine; Lv; liver; Pan, pancreas.

*Shp* is expressed in many metabolic tissues, including the intestine, pancreas, and kidney. Therefore, it is still unclear that hepatic SHP plays a major role in the observed liver phenotypes. Therefore, we generated liver-specific *Shp*^*−/−*^ mice (*LvShp*^*−/−*^) using floxed *Shp* mice (*Shp*^*fl/fl*^) (16) and *Alb-Cre* mice (Jackson Laboratory). Liver-specific *Shp* gene deletion was confirmed by RT–qPCR analysis (Fig. 7A). To define the role of hepatic SHP in NASH development, *LvShp*^*−/−*^ mice were fed a WD for 6 months, and their hepatic inflammatory gene expression was compared with those from *Alb-Cre* or *Shp*^*fl/fl*^(*fShp*) mice. The *LvShp*^*−/−*^ mice displayed lower hepatic TG and cholesterol accumulation compared with the control mice (Fig. 7B) and lower inflammatory gene expression (Fig. 7C and supplemental Fig. S3A) with lower body weight gain (supplemental Fig. S3B) upon WD feeding than in either *fShp* mice or *Alb-Cre* mice. The expressions of genes involved in fibrogenesis were also significantly downregulated in the livers of *LvShp*^*−/−*^ mice compared with those from *fShp* and *Alb-Cre* mice fed the WD (Fig. 7C and supplemental Fig. S3A). As hepatic TG and cholesterol levels were higher in the *Alb-Cre* mice than in the *fShp* mice and gene expression was comparable between the two control groups, we thereafter primarily used *fShp* mice as controls against the *LvShp*^*−/−*^ mice. In addition, ROS production in *LvShp*^*−/−*^ mice fed the WD was reduced compared with that in the WT mice (Fig. 2D), which was likely because of the reduced mRNA expression of NADPH oxidase genes (supplemental Fig. S4A). Serum lipoprotein profiles and their TG and cholesterol contents were similar to those of whole-body *Shp*^*−/−*^ mice (supplemental Fig. S4B).

**Fig. 8:**
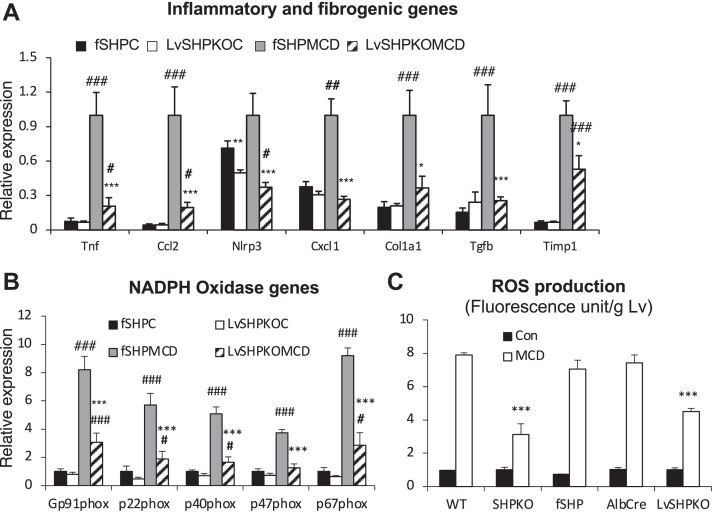
Liver-specific *Shp* deletion was responsible for the protection from MCD diet-induced NASH progression. fSHP mice and LvShpKO mice were fed MCD diet for 1 month, and their livers were collected for RNA isolation (*n* = 7–9). Another group of mice was used to determine hepatic ROS production after dihydroethidium (DHE) i.p. injection (*n* = 5). mRNA levels of genes in inflammation/fibrogenesis pathways (A) and NADPH oxidase function (B) were quantified using qPCR analysis. C: ROS production was presented as fluorescence units from liver lysates after normalization by wet liver weight. Student’s *t*-test was performed for statistical analysis, and the results were presented as indicated for Fig. 1.

We also challenged the *LvShp*^*−/−*^ mice with the MCD diet to explore the NASH phenotype further. After the 1 month challenge, strong hepatic fat accumulation was evident by Oil Red O staining in *fShp* mice but not in *LvShp*^*−/−*^ mice, as observed in whole-body knockout animals (supplemental Fig. S5A). In addition, hepatic genes involved in the inflammatory response and fibrogenesis were significantly downregulated, and F4/80-positive cells were reduced in the *LvShp*^*−/−*^ liver compared with those in the *fShp* liver (Fig. 8A and supplemental Fig. S5B). As observed in the whole-body knockout mice, oxidative stress also decreased in the *LvShp*^*−/−*^ mice, as evidenced by their NADPH gene expression and ROS production (Fig. 8B, C). These results strongly suggest that hepatic SHP is a driving force for the development of the NASH phenotype induced by the WD, and its deletion protects mice from this metabolic disorder.

## Discussion

Orphan nuclear hormone receptor *Shp* was cloned approximately two decades ago as a gene encoding a protein interacting with the constitutive androstane receptor, another nuclear hormone receptor involved in xenobiotic and endobiotic stress, in a yeast two-hybrid screening (42). Subsequently, it was reported that SHP interacts with other transcription factors, including several nuclear hormone receptors, to exert its transcriptional repression activity (17, 42, 43, 44, 45). Among its gene regulatory activities, the regulation of *Cyp7a1*, which encodes a rate-limiting enzyme in the conversion of cholesterol to BAs, gene expression has been recognized and widely discussed (5, 6). The generation of mutant mice lacking or overexpressing the *Shp* gene not only confirmed the importance of the *Cyp7a1* regulatory role in BA metabolism but also extended the understanding of the underlying metabolic processes (7, 8, 10, 14). These studies revealed the important role of SHP in hepatic lipid homeostasis, where its overexpression leads to fat accumulation, but deletion leads to reduced fat accumulation. Our study identified a strong association of SHP with the inflammatory response, an important step in the development of NASH.

Earlier studies claimed that deletion of SHP exacerbated hepatic inflammatory responses (19, 20, 21). Many studies, including ours, have reported that deletion of *Shp* attenuates body weight gain and hepatic lipid accumulation induced by high-calorie diets in mice. In addition, our BeadChip array analysis showed that the expression of important inflammatory genes was downregulated in *Shp*^*−/−*^ mice fed the WD. Thus, the reduced hepatic inflammatory responses can be attributed to a secondary effect because of a reduction in fat accumulation, which has been suggested as a two-hit theory for NASH development (2). To determine this question, we introduced *Shp* deletion in *ApoE*-null mice, where hepatic inflammation was induced by the WD without fat accumulation (23). In this study, we identified that a 1 week WD regimen was sufficient to induce inflammatory responses in the *ApoE*-null mice without hepatic fat accumulation. In this short-term diet challenge, *Shp* deletion ameliorated inflammatory responses without significant changes in the TG or cholesterol levels compared with those in the *ApoE*^*−/−*^ mice, suggesting an intrinsic effect of SHP on diet-induced inflammation. In an earlier study, plasma VLDL cholesterol levels were found to be linked to hepatic inflammation (38). As reported, the VLDL fractions in the WT and *ApoE*^*−/−*^ mice increased (supplemental Fig. S2), and their total cholesterol contents (area under the curve) increased significantly when they were fed the WD (Fig. 4). Although the exact mechanism of induction of hepatic inflammation by plasma VLDL cholesterol remains unidentified, our conclusion about the roles of TG and cholesterol in the development of hepatic inflammation strongly corroborates with observations. Mari *et al.* (31) also claimed that the accumulation of free cholesterol in the mitochondria of hepatocytes sensitizes the steatotic liver to inflammatory cytokines via mitochondrial glutathione depletion. The inhibition of this proposed pathway may have ameliorated NASH development in our *Shp*^*−/−*^ mice, as evidenced by the structures and gene expressions of mitochondria and hepatic cholesterol levels with WD feeding (Fig. 3 and (10)). The attenuation of the second insult by *Shp* deletion was reconfirmed in the MCD diet challenge. Although a similar NASH pathology is presented by the two diets, the proposed underlying mechanisms for disease development are different (46, 47). *Shp* deletion decreased hepatic cholesterol but not plasma cholesterol in the MCD diet challenge, but it manifested an attenuation of NASH development (Fig. 5). Based on earlier observations that free cholesterol loading on hepatocytes or the dietary cholesterol component is sufficient to trigger both hepatic TG accumulation and inflammation (31, 39), we suggest that the reduction of hepatic cholesterol by *Shp* deletion may play a critical role in the attenuation of the NASH phenotype manifested by the two diet challenges. Induction of *Cyp7a1* gene expression by *Shp* gene deletion plays a role in the observed hepatic cholesterol levels in the mice. In addition, the protection was reproduced in the liver-specific null mice, which showed that the role of SHP in hepatic clearance of cholesterol to BA may be a crucial player in NASH development.

The current study suggested that the regulatory role of hepatic SHP in *Cyp7a1* gene expression may play a role in the NASH phenotype. SHP deletion protects mitochondria by preventing cholesterol accumulation, thus minimizing ROS production. The suggested metabolic role of *Cyp7a1* is partially supported by an earlier report in which overexpression of *Cyp7a1* reversed strong NASH phenotypes observed in MCD diet-challenged *Cyp7a1*^*−/−*^ mice (48). In the accompanying study, we identified that another mechanism associated with the regulatory role of SHP in BA metabolism was involved in NASH development. The modified BA profile by induction of *Cyp7a1* in *Shp*^*−/−*^ mice changes the gut flora and their metabolites. Cohousing of *Shp*^*−/−*^ mice with WT mice disrupted the protected NASH phenotypes manifested by the null mice by modification of gut microbiota. Two specific bacterial products were suggested to be responsible for the NASH progression. Reduction of these products in the circulatory system was associated with the observed protection in *Shp*^*−/−*^ mice. Therefore, *Shp* appears to control multiple steps in the development of NASH. Of note, cohousing also disrupted the reduction of hepatic cholesterol in *Shp*^*−/−*^ mice but maintained the induction of *Cyp7a1* gene expression. Although the underlying mechanism is unknown, changes in the gut microbiome may indirectly induce hepatic cholesterol levels in null animals independent of *Cyp7a1* gene expression. Orphan nuclear receptor SHP could serve as a pharmaceutical target for the efficient treatment of NASH.

## Data Availability

All data are contained within this article.

## Supplemental data

This article contains supplemental data.

## Conflict of interest

The authors declare that they have no conflicts of interest with the contents of this article.
