# Supplementary material for: Deletion of hepatic small heterodimer partner ameliorates development of nonalcoholic steatohepatitis in mice
Source: J Lipid Res. 2023 Oct 10;64(11):100454. doi: 10.1016/j.jlr.2023.100454 (PMC10665942; doi:10.1016/j.jlr.2023.100454)
Supplement: Supplemental Tables S1 and S2 [file mmc1.pdf]

Gene Symbol	WTCD	KOCD	WTWD	KOWD	p value (WTWD vs KOWD)
Il1b	33.7±4.9	52.5±4.8	82.4±8.7	48±5.5	0.024
Cxcl1	91.4±6.6	58.2±7.5	314.6±19.6	88.1±7.1	0.015
Ccl2	-0.4±3.3	2.6±3.5	21±4.9	-0.9±3.2	0.024
Ccl3	0.8±3.1	0.2±3.1	30.4±5.3	2.8±3.0	0.000
Ccl4	19.1±4.1	8.7±3.9	141.3±10.0	14.8±4.2	0.000
Timp1	3.1±4.9	0.8±2.4	87.1±8.8	13±3.1	0.086
Col1a1	7.9±3.6	4.8±3.3	75.4±9.4	11.1±4.0	0.005

Supplemental Table S1. Inflammatory gene expression in livers of WT and SHPKO mice fed chow or WD

Inflammatory genes differentially regulated between livers of WT and SHPKO mice fed WD were identified from an earlier Illumina BeadChip array result (20). The values are presented as means ± SEM (n=4).

Gene Symbol	WTCD	KOCD	WTWD	KOWD	p value (WTWD vs KOWD)
Cp	4684.5±2934	4693±230	5648.3±332	42678±193	0.043
Gpx3	146.3±12.5	183.2±12.0	185.7±16.1	130.8±12.2	0.032
Gpx4	799.5±44.9	815.5±35	1321.3±59	757.6±37.3	0.000
Gsta1	12.7±3.8	-0.3±2.6	629.2±28.2	38.5±4.5	0.001
Gsta2	164.8±16.4	68.4±6.1	705.8±41.1	105.5±8.2	0
Mgst3	85.4±7.8	56.4±6.5	234.6±15.7	63±6.5	0
Txn2	372.1±18.7	380.7±18.4	267.1±17.5	376±20.6	0.013

Supplemental Table S2. Antioxidant gene expression in livers of WT and SHPKO mice fed chow or WD

Antioxidant genes differentially regulated between livers of WT and SHPKO mice fed WD were identified from an earlier Illumina BeadChip array result (20). The values are presented as means ± SEM (n=4).

Supplemental Figure S1

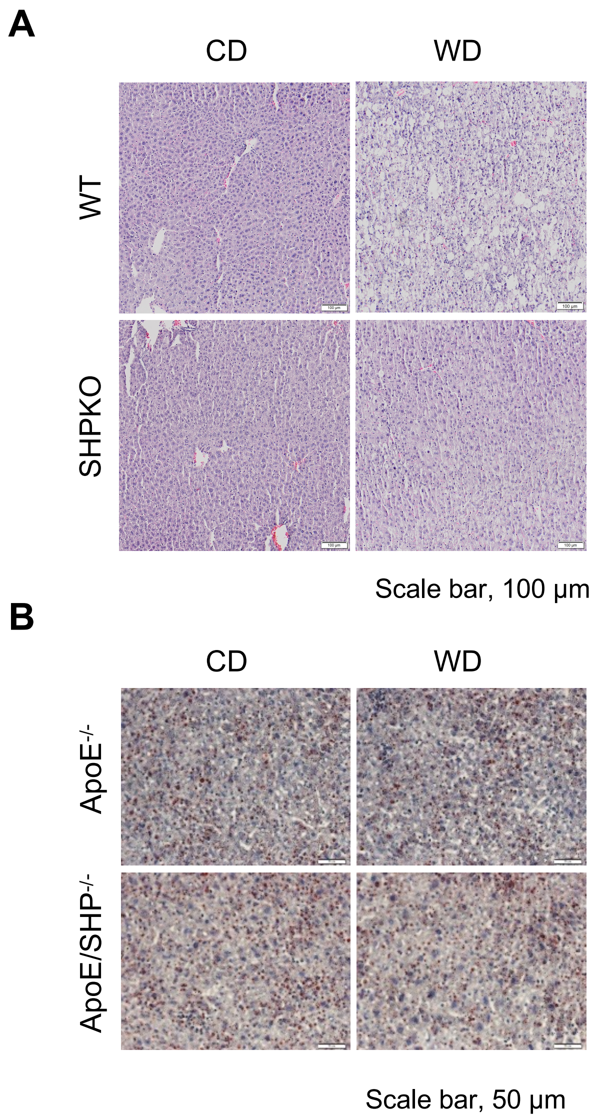

Supplemental Figure S1. H&E staining and Oil-red O staining with liver sections from WT and Shp$^{-/-}$ mice, ApoE$^{-/-}$ and ApoE$^{-/-}$/Shp$^{-/-}$ mice, respectively.

A) Representative H&E-staining sections of livers from WT and Shp-/- mice fed WD for 6 months, which were evaluated for NAFLD activity scores in main Fig. 1C. B) Cryosections of livers from ApoE$^{-/-}$ and ApoE$^{-/-}$/Shp$^{-/-}$ mice fed chow or 1-week WD regimen were processed for Oli-red O staining and visualized with a microscope (x100).

Supplemental Figure S2

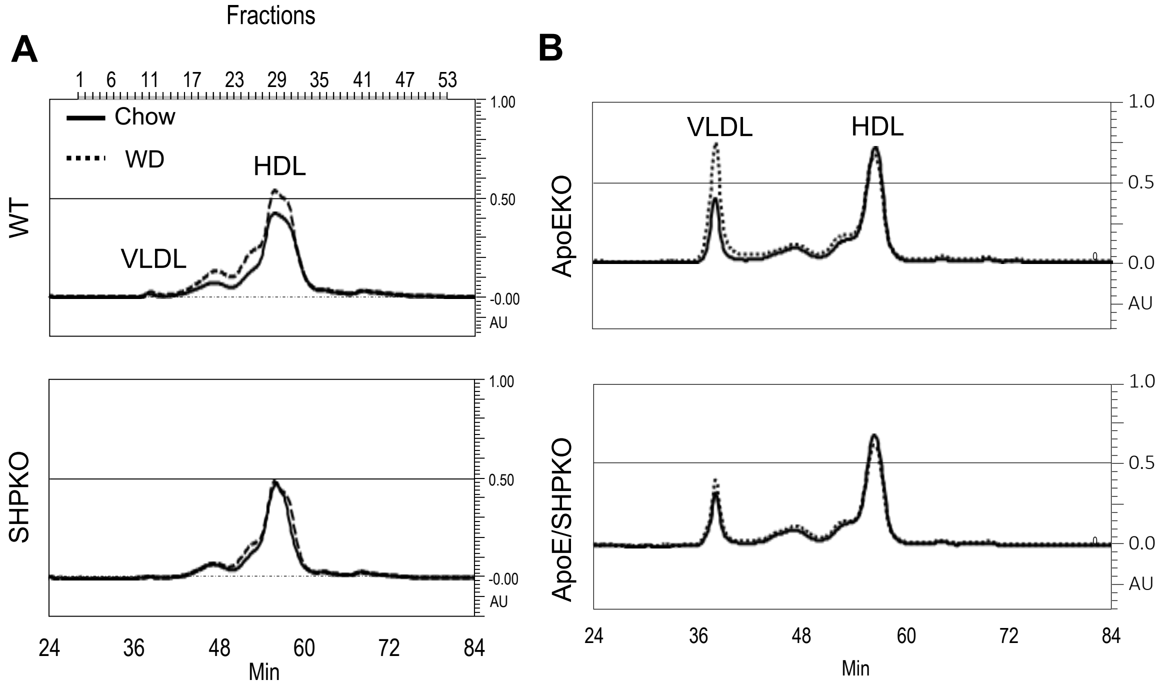

Supplemental Figure S2. Plasma lipid profiles identified by FPLC analysis in WT, SHP$^{-/-}$, ApoE$^{-/-}$, and ApoE/SHP$^{-/-}$ mice fed chow or WD.

Plasma samples obtained experimental animals were analyzed using a fast protein liquid chromatography as described in the main methods. Absorbance curve of individual fraction from each group (n=5) was presented with their elution times.

Supplemental Figure S3

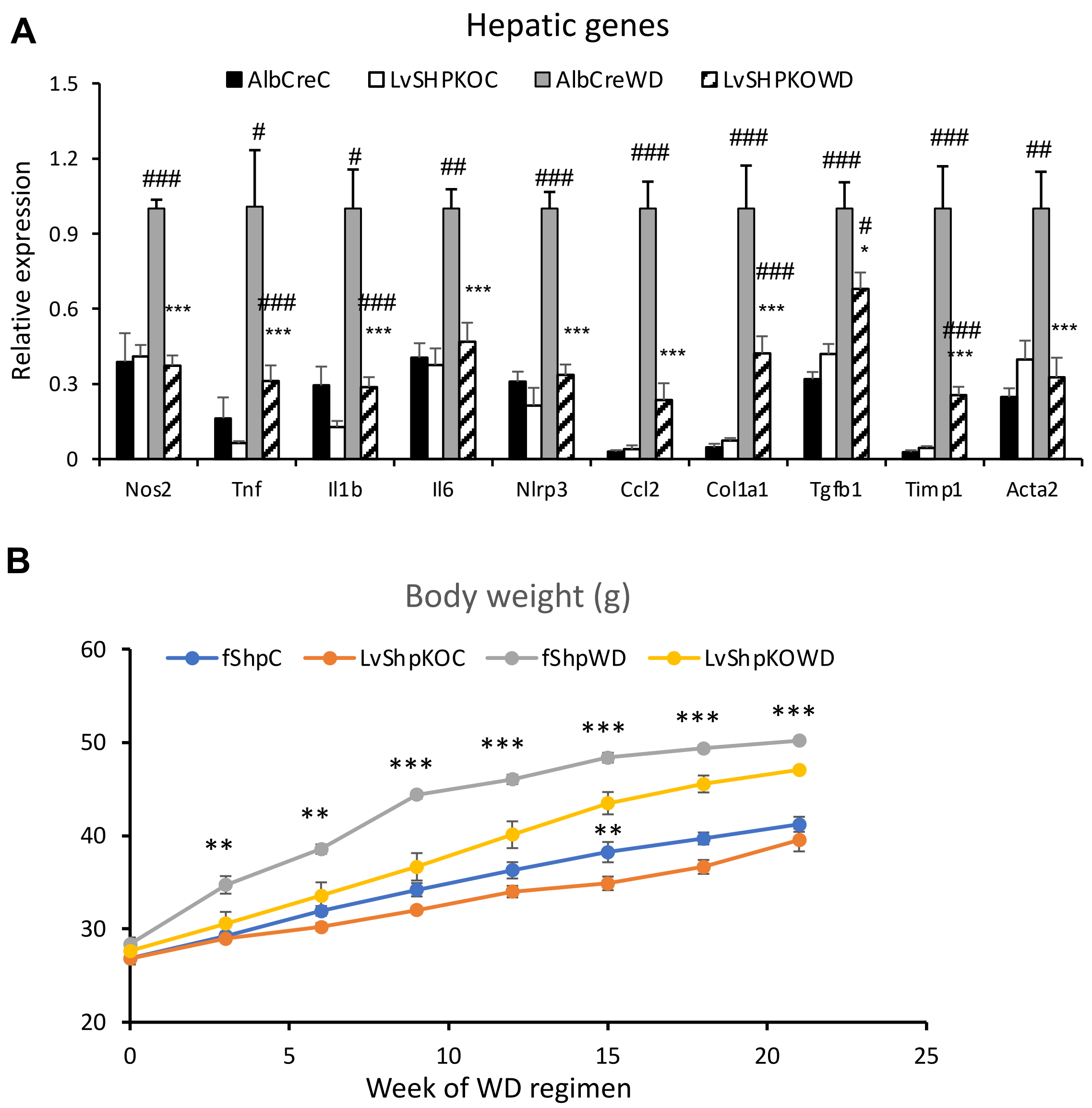

Supplemental Figure S3. Hepatic gene expression and body weight changes in liver-specific SHPKO animals on WD regimen. (A) Hepatic gene expression in Alb-Cre and LvSHPKO mice fed chow or WD was quantified using qPCR analysis. (B) Body weight was monitored during WD regimen on fSHP and LvSHPKO mice and presented as mean ± SEM (n=6-7). *, comparison between genotypes; #, comparison between two diets. * or #, $p < 0.05$; ** or ##, $p < 0.01$, *** or ###, $p < 0.005$

Supplemental Figure S4

A

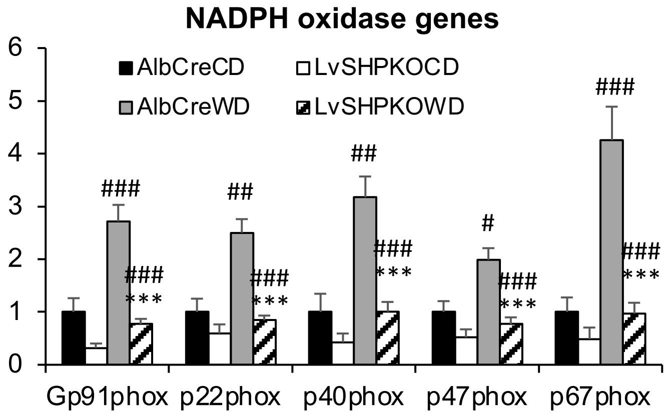

B

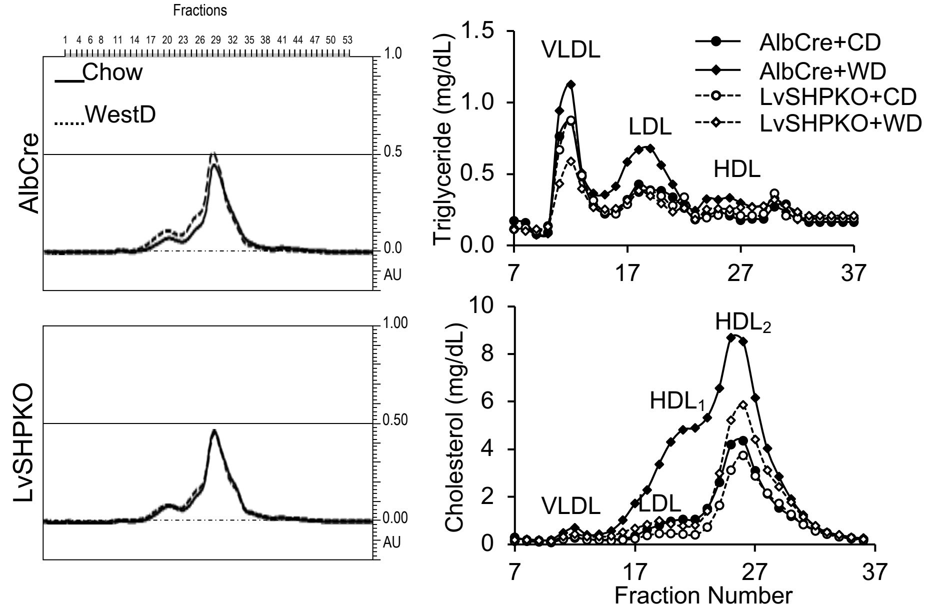

Supplemental Figure S4. mRNA expression of NADPH oxidase genes and plasma lipid profiles in LvSHPKO mice.
(A) Expression of indicated NADPH oxidase genes was assessed using qPCR analysis and presented as means ± SEM. n=4 to 9. Student t-test was performed to obtain statistical values. #; comparison with CD, *; comparison with AlbCre counterparts. Three symbols represent p value < 0.005, two symbols p value < 0.01, one symbol p value < 0.05
(B) FPLC analysis was performed to present plasma lipoprotein profiles in AlbCre and LvSHPKO mice. Plasma lipoprotein absorbance curve for AlbCre and LvSHPKO mice was presented (left panel). TG (top) and cholesterol (bottom) levels of fractionated plasma lipoproteins were quantified and plotted as curve graphs (right panel).

Supplemental Fig. S5

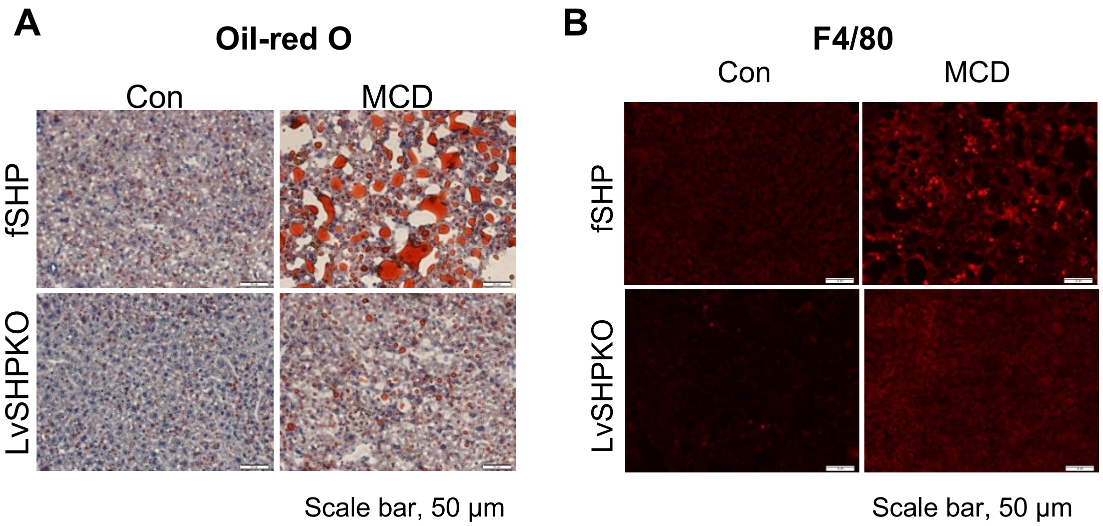

Supplemental Figure S5. Assessment of NASH development in LvSHPKO mice upon MCD diet challenge.
(A) Oil-red O staining was performed with liver sections from fSHP and LvSHPKO mice fed control or MCD diet. (B) Immunofluorescence was carried out using F4/80 antibodies on liver sections of the animals.
