# Supplementary material for: Deletion of hepatic small heterodimer partner ameliorates development of nonalcoholic steatohepatitis in mice
Source: J Lipid Res. 2023 Oct 10;64(11):100454. doi: 10.1016/j.jlr.2023.100454 (PMC10665942; doi:10.1016/j.jlr.2023.100454)
Supplement: Supplemental Figures S1–S5 [file mmc2.pdf]

Supplemental Figure S1

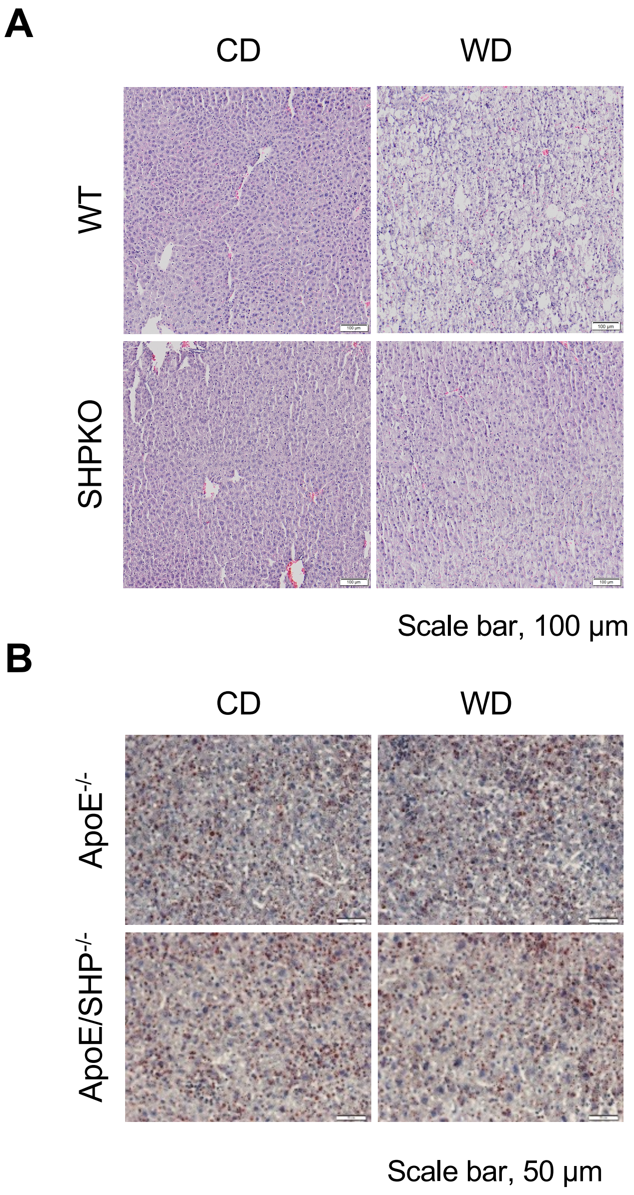


**Supplemental Figure S1. H&E staining and Oil-red O staining with liver sections from WT and $Shp^{-/-}$ mice, $ApoE^{-/-}$ and $ApoE^{-/-}/Shp^{-/-}$ mice, respectively.**
A) Representative H&E-staining sections of livers from WT and Shp-/- mice fed WD for 6 months, which were evaluated for NAFLD activity scores in main Fig. 1C. B) Cryosections of livers from $ApoE^{-/-}$ and $ApoE^{-/-}/Shp^{-/-}$ mice fed chow or 1-week WD regimen were processed for Oli-red O staining and visualized with a microscope (x100).

Supplemental Figure S2

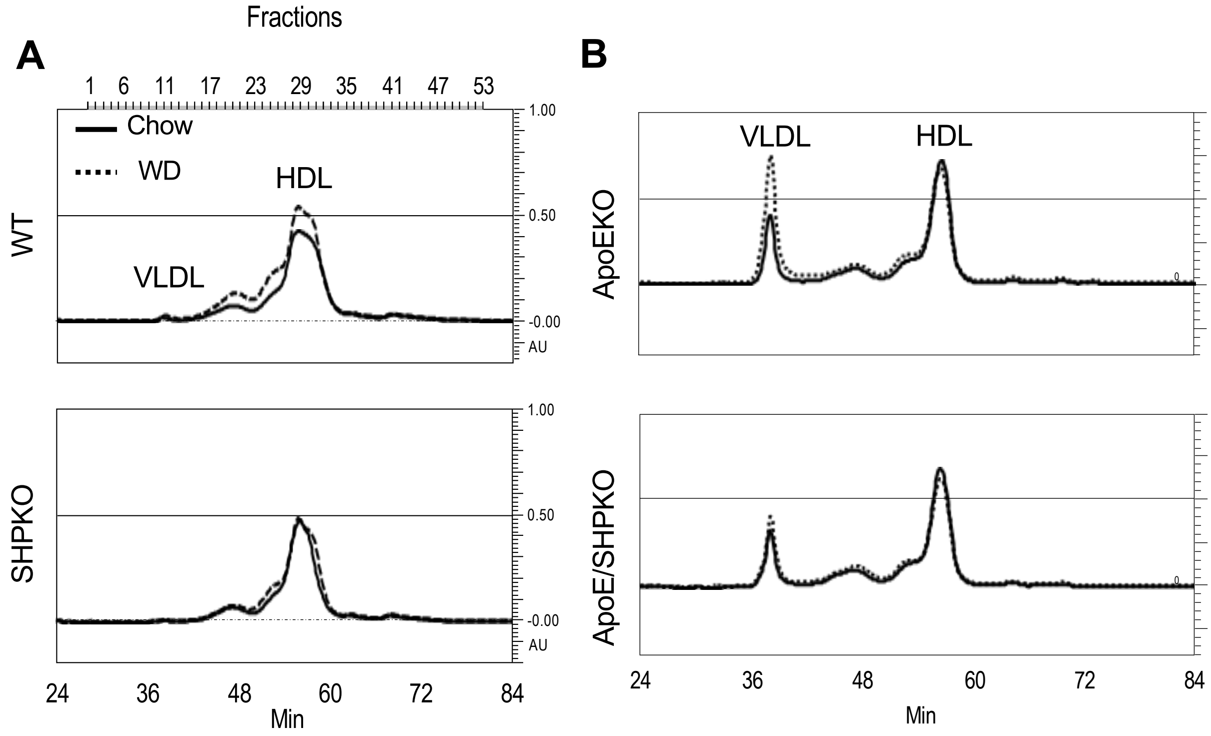


**Supplemental Figure S2. Plasma lipid profiles identified by FPLC analysis in WT, SHP$^{-/-}$, ApoE$^{-/-}$, and ApoE/SHP$^{-/-}$ mice fed chow or WD.**

Plasma samples obtained experimental animals were analyzed using a fast protein liquid chromatography as described in the main methods. Absorbance curve of individual fraction from each group (n=5) was presented with their elution times.

Supplemental Figure S3

A

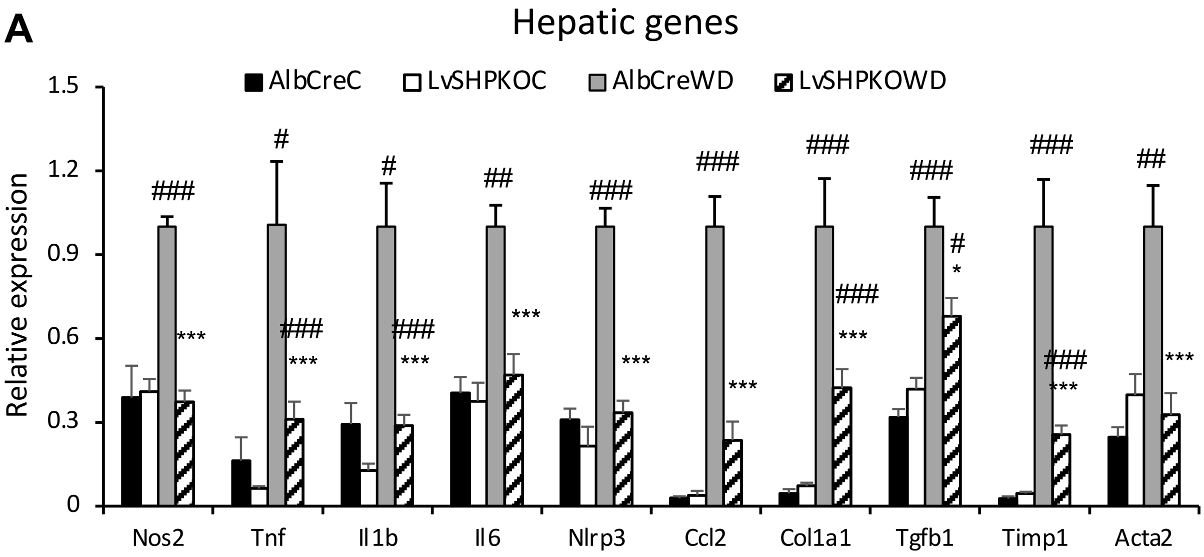


B

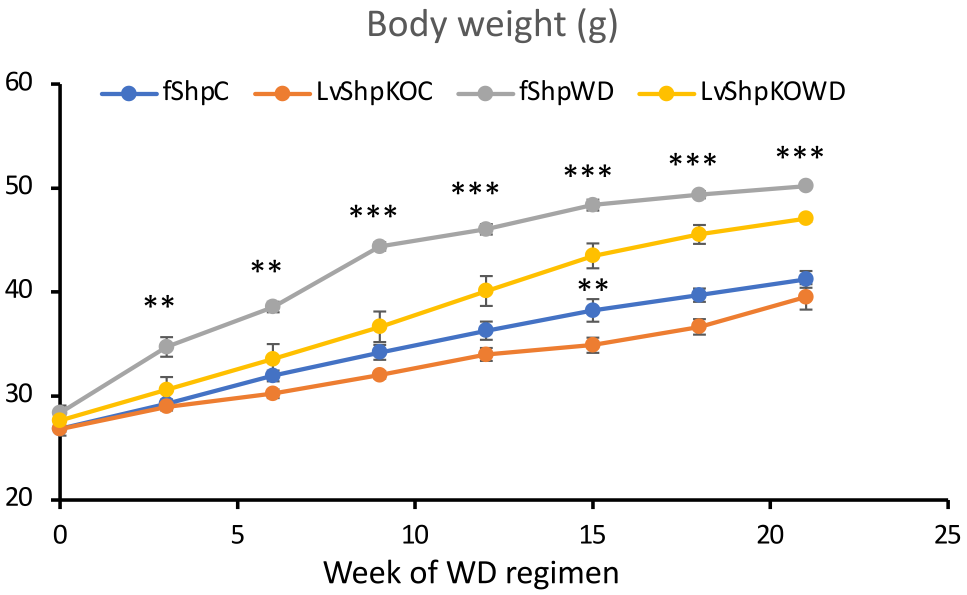


**Supplemental Figure S3. Hepatic gene expression and body weight changes in liver-specific SHPKO animals on WD regimen.** (A) Hepatic gene expression in Alb-Cre and LvSHPKO mice fed chow or WD was quantified using qPCR analysis. (B) Body weight was monitored during WD regimen on fSHP and LvSHPKO mice and presented as mean ± SEM (n=6-7). *, comparison between genotypes; #, comparison between two diets. * or #, $p < 0.05$; ** or ##, $p < 0.01$, *** or ###, $p < 0.005$

Supplemental Figure S4

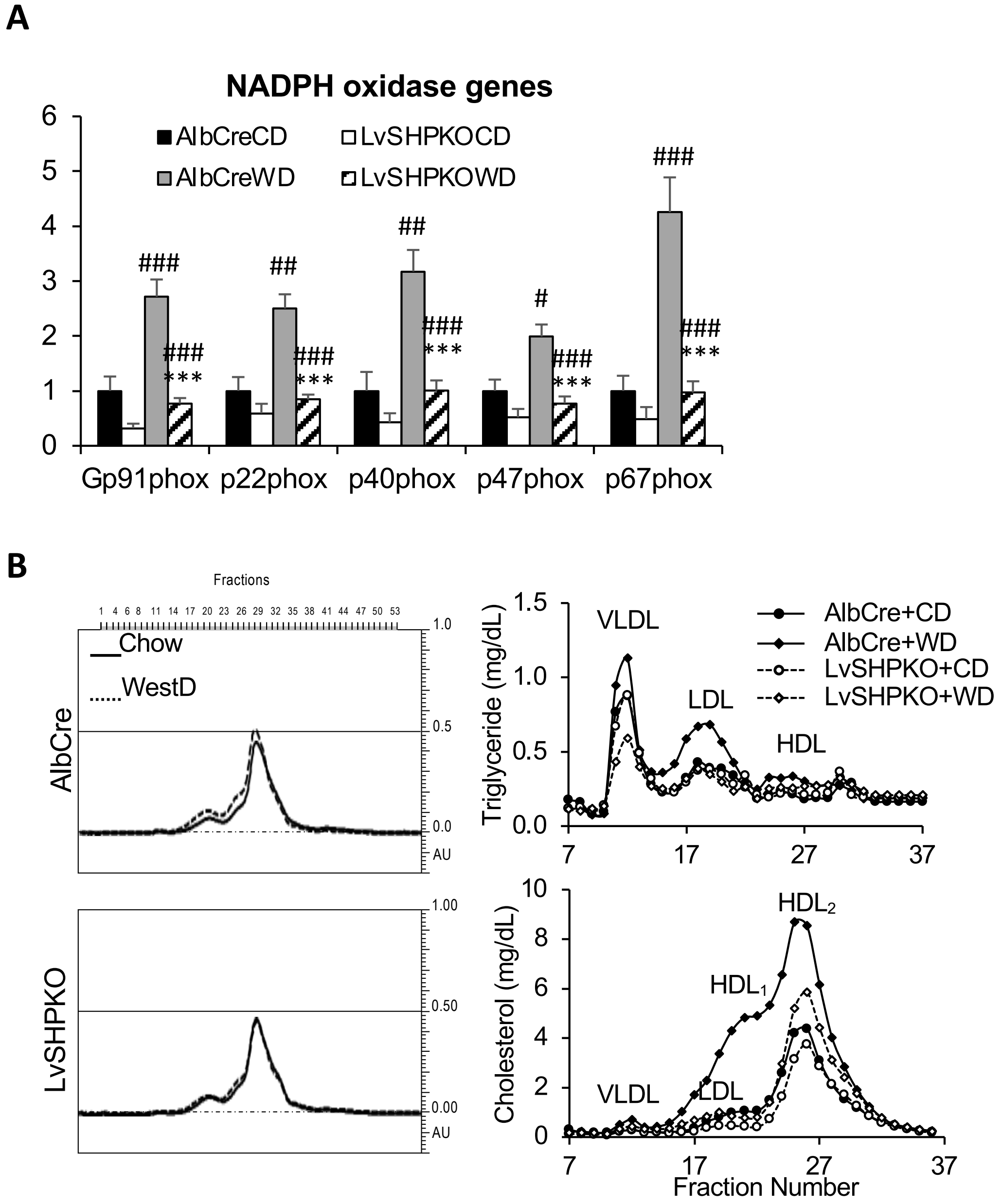


**Supplemental Figure S4. mRNA expression of NADPH oxidase genes and plasma lipid profiles in LvSHPKO mice.**

(A) Expression of indicated NADPH oxidase genes was assessed using qPCR analysis and presented as means ± SEM. n=4 to 9. Student t-test was performed to obtain statistical values. #; comparison with CD, *; comparison with AlbCre counterparts. Three symbols represent p value < 0.005, two symbols p value < 0.01, one symbol p value < 0.05 (B) FPLC analysis was performed to present plasma lipoprotein profiles in AlbCre and LvSHPKO mice. Plasma lipoprotein absorbance curve for AlbCre and LvSHPKO mice was presented (left panel). TG (top) and cholesterol (bottom) levels of fractionated plasma lipoproteins were quantified and plotted as curve graphs (right panel).

Supplemental Fig. S5

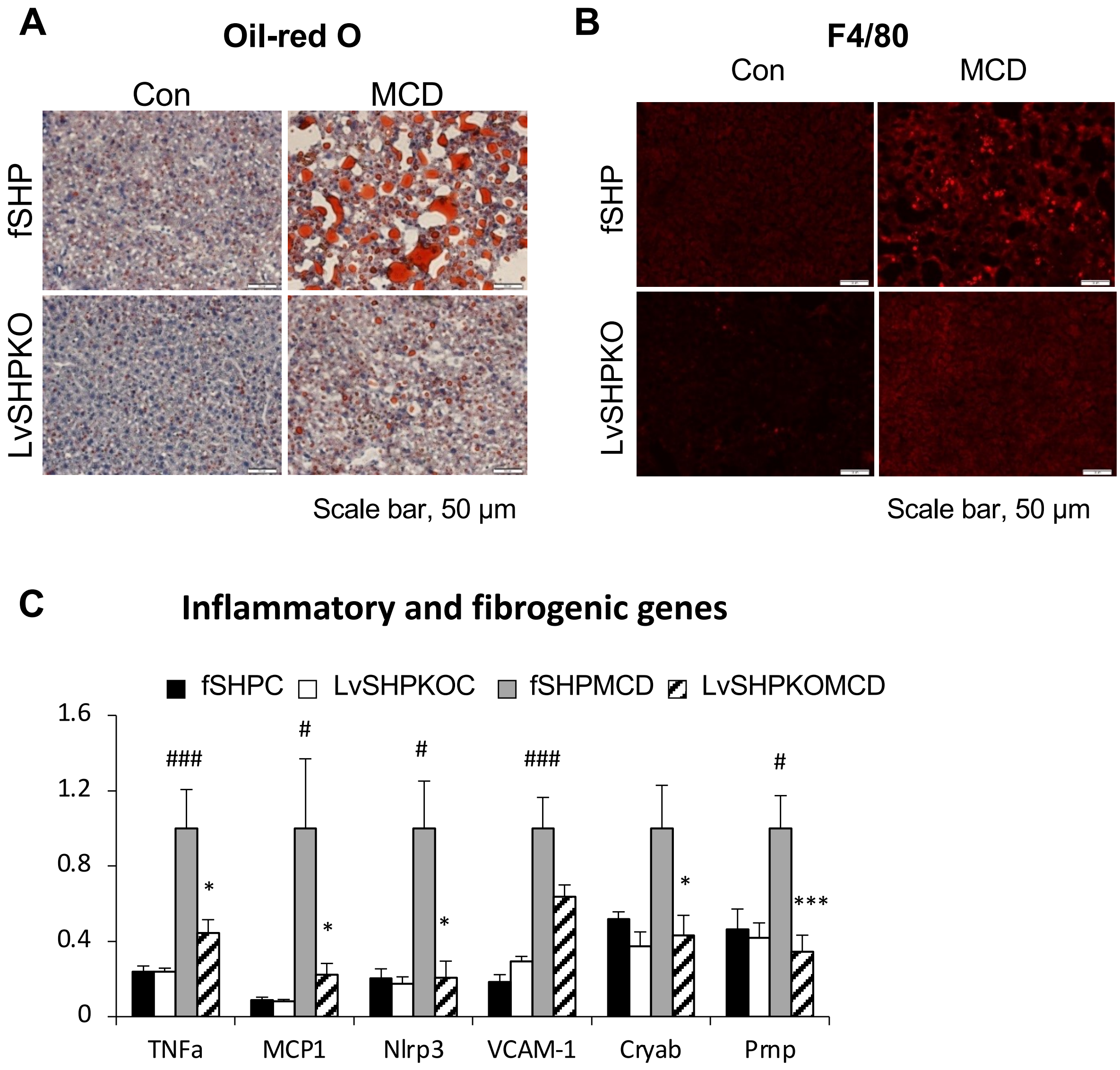


**Supplemental Figure S5. Assessment of NASH development in LvSHPKO mice upon MCD diet challenge.**

(A) Oil-red O staining was performed with liver sections from fSHP and LvSHPKO mice fed control or MCD diet. (B) Immunofluorescence was carried out using F4/80 antibodies on liver sections of the animals. (C) Hepatic inflammation and fibrogenesis were assessed by qPCR analysis for the LvSHPKO mice fed MCD diet.
